# Crystal structure of new formamidinium triiodide jointly refined by single-crystal XRD, Raman scattering spectroscopy and DFT assessment of hydrogen-bond network features

**DOI:** 10.1107/S2056989021005673

**Published:** 2021-06-08

**Authors:** Artem A. Ordinartsev, Andrey A. Petrov, Konstantin A. Lyssenko, Andrey V. Petrov, Eugene A. Goodilin, Alexey B. Tarasov

**Affiliations:** a Laboratory of New Materials for Solar Energetics, Faculty of Materials Science, Lomonosov Moscow State University, 119991 Moscow, Russian Federation; b Department of Chemistry, Lomonosov Moscow State University, 119991 Moscow, Russian Federation; c Institute of Chemistry, Saint-Petersburg State University, 198504, Saint-Petersburg, Russian Federation; d Institute of General and Inorganic Chemistry, 119991, Moscow, Russian Federation

**Keywords:** polyiodides, formamidinium, formamidinium triiodide, reactive polyiodide melts, Raman spectroscopy, DFT, crystal structure

## Abstract

A novel triiodide phase of the formamidinium cation, CH_5_N_2_^+^·I_3_^−^, crystallizes in the triclinic space group *P*  at a temperature of 110 K. The structure consists of two independent isolated triiodide ions located on inversion centers. The centrosymmetric character of I_3_^−^ was additionally confirmed by the observed pronounced peaks of symmetrical oscillations of I_3_^−^ at 115–116 cm^−1^ in Raman scattering spectra. An additional structural feature is that each terminal iodine atom is connected with three neighboring planar formamidinium cations by N—H⋯I hydrogen bonding with the N—H⋯I bond length varying from 2.81 to 3.08 Å, forming a deformed two-dimensional framework of hydrogen bonds. A Mulliken population analysis showed that the calculated charges of hydrogen atoms correlate well with hydrogen-bond lengths. The crystal studied was refined as a three-component twin with domain ratios of 0.631 (1):0.211 (1):0.158 (1).

## Abstract 2

A novel triiodide phase of the formamidinium cation, CH_5_N_2_^+^·I_3_^−^, crystallizes in the triclinic space group *P* 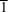 at a temperature of 100 K. The structure consists of two independent isolated triiodide ions located on inversion centers. The centrosymmetric character of I_3_^−^ was additionally confirmed by the observed pronounced peaks of symmetrical oscillations of I_3_^−^ at 115–116 cm^−1^ in Raman scattering spectra.

## Chemical context   

Polyiodides are a large class of compounds with organic and inorganic cations and a great diversity of anion shapes varying from simple linear I_3_^−^ up to I_29_^3–^ complexes (Svensson & Kloo, 2003 ▸). Such a great diversity of cations and anions allows one to tune the chemical and physical properties of the target compounds. Consequently, polyiodides have attracted great inter­est for a wide set of applications, such as dye-sensitized solar cells (DSSC) (O‘Regan & Grätzel, 1991 ▸; Jeon *et al.*, 2011 ▸), different electrochemical devices (Weinstein *et al.*, 2008 ▸; Weng *et al.*, 2017 ▸) and light-polarizing materials (Kahr *et al.*, 2009 ▸).

Another recently proposed prospective application of polyiodides is to use liquid methyl­ammonium and formamidinium polyiodides as a precursor for the fabrication of light-absorbing materials for perovskite solar cells (Petrov, Belich *et al.*, 2017 ▸). Whereas the application of formamidinium polyiodides was shown to be successful for scalable fabrication of solar cells with efficiencies over 17% (Turkevych *et al.*, 2019 ▸), the structures of formamidinium polyiodides have not been studied so far.

In this work, we investigated the features of the new structure of the single-crystalline CH_5_N_2_^+^·I_3_^−^ (**I**, FAI_3_) phase by means of Raman scattering spectroscopy and DFT calculations.
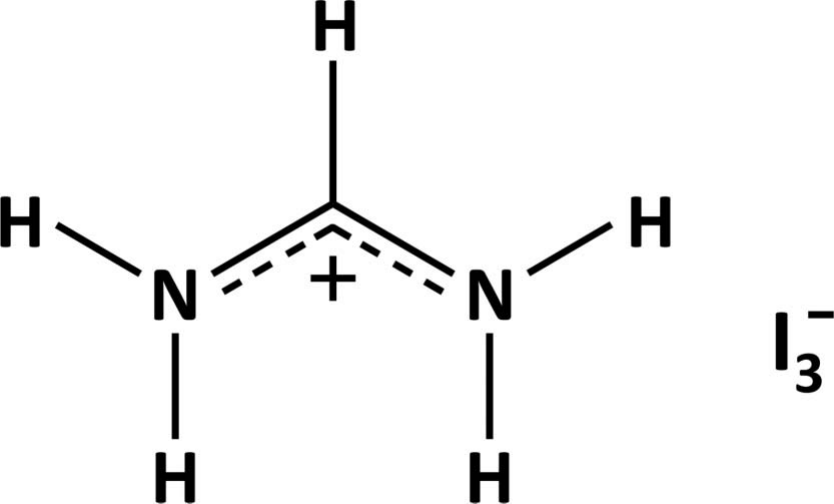


## Structural commentary   

**Figure 1:**
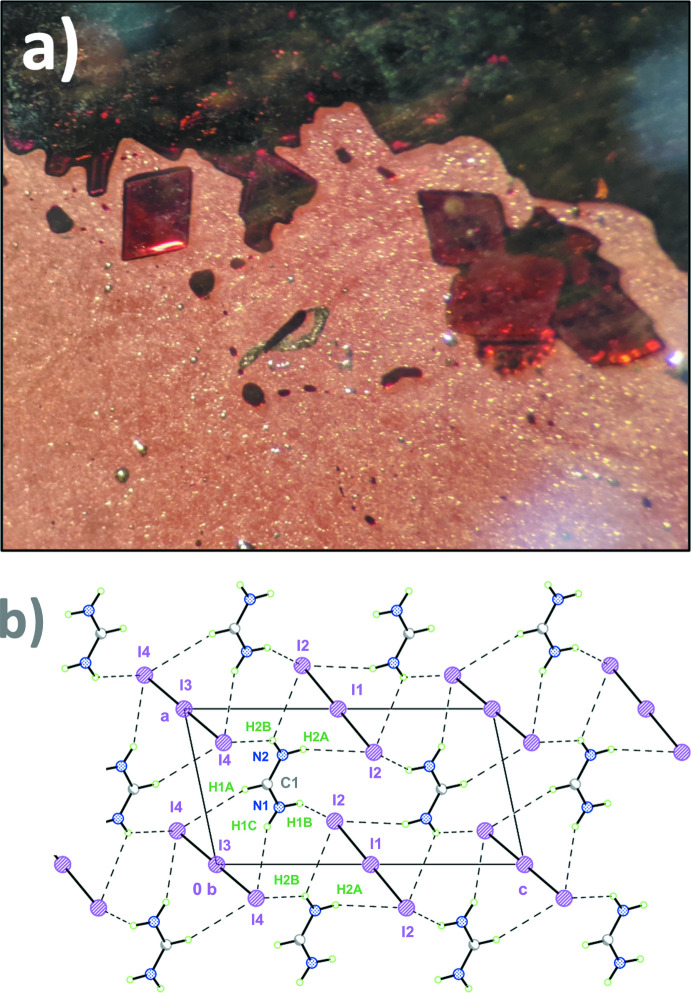
(*a*) FAI_3_ single crystals used for the determination of the crystal structure. (*b*) FAI_3_ crystal structure with solid lines indicating covalent bonding and dashed lines indicating the inter­molecular hydrogen bonds.

Dark-red transparent rhombic-shaped single crystals (Fig. 1 ▸ *a*) were obtained by slow heating of preliminary powdered stoichiometric FAI/I_2_ (FA = CH_5_N_2_^+^) mixture up to 355–358 K. Such a temperature range allowed us to obtain well-shaped single crystals as a result of recrystallization from the liquid state near its melting point, which was determined to be *T_m_* = 360 K by visual thermal analysis.

FAI_3_ was found to crystallize in a triclinic unit cell, space group *P* 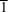. The structure (Fig. 1 ▸ *b*) consists of two types of isolated centrosymmetric triiodide ions (*D*_∞*h*_ point symmetry) located on centers of inversion. Therefore, only centrosymmetric I_3_^−^ anions are present, which is rare for structures with relatively small cations such as formamidinium (Svensson & Kloo, 2003 ▸; Gabes & Gerding, 1972 ▸). For instance, in the CsI_3_ crystal structure, the I_3_^−^ anion is asymmetric (*C_s_* point symmetry) with ∠(I—I—I) = 178° (Runsink *et al.*, 1972 ▸). For a similar CH_3_NH_3_I/I_2_ system, the CH_3_NH_3_I_3_ structure was not isolated (Petrov *et al.*, 2019 ▸).

**Figure 2:**
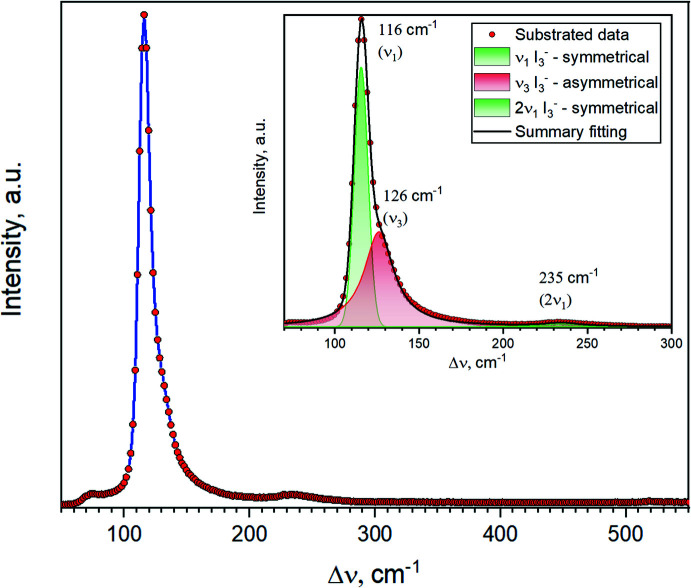
Raman spectrum of a transparent single crystalline plate of FAI_3_. Laser wavelength, 633 nm; laser power, 20 mW; accumulation time, 1 min. The insert demonstrates the results of spectroscopic analysis.

The centrosymmetric character of the I_3_^−^ anions in FAI_3_ was confirmed by Raman scattering spectroscopy. The Raman spectrum recorded using a 633 nm laser (Fig. 2 ▸) contains a pronounced peak of ν_1_(I_3_^−^) symmetrical oscillations at 116 cm^−1^ with an additional 235 cm^−1^ 2ν_1_(I_3_^−^) peak and an asymmetrical ν_3_(I_3_^−^) component at 126 cm^−1^ (Svensson & Kloo, 2003 ▸). The latter might be observed because of the presence of two types of I_3_^−^ units in the structure with different environments. In addition, no splitting of symmetric oscillations is observed in the Raman spectrum because of the very small difference between the two types of I_3_^−^ in the structure.

The first of the two types of I_3_^−^ anions in FAI_3_ [*d*(I1—I2) = 2.9165 (14) Å] is connected with three neighboring planar formamidinium cations by N—H⋯I hydrogen bonding with the bond length varying from 2.81 to 3.08 Å (Table 1 ▸), which is similar to the distances in formamidinium iodide (Petrov, Goodilin *et al.*, 2017 ▸) as well as in other polyiodides (Petrov *et al.*, 2019 ▸; Said *et al.*, 2006 ▸; van Megen & Reiss, 2013 ▸). The second type of I_3_^−^ anions are connected by two N—H⋯I hydrogen bonds of slightly reduced length and a relatively less strong C—H⋯I hydrogen bond [*d*(CH1*A*⋯I4) = 3.03 Å]. Such a difference, however, does not change significantly the distance between the central and terminal iodine atoms [2.9165 (14) *vs* 2.9209 (14) Å].

A Mulliken population analysis showed that charges of hydrogen atoms forming a single hydrogen bond with a terminal iodine atom is +0.152 for atom H1*A*, which is connected with carbon, whereas it is +0.171 for atom H2*A* connected with nitro­gen (Table 1 ▸), which correlates with the corresponding hydrogen-bond lengths [*d*(CH1*A*⋯I4) = 3.03 Å *vs d*(NH2*A*··I2) = 2.81 Å]. For the H2*B* hydrogen atom, the high atomic charge (+0.189) is distributed by two hydrogen bonds. An analysis of the Bader atomic charges for the isolated cation also shows a higher charge for the H2*A* atom in comparison with H1*A* (Table 2 ▸), which correlates well with the hydrogen-bond lengths.

**Figure 3:**
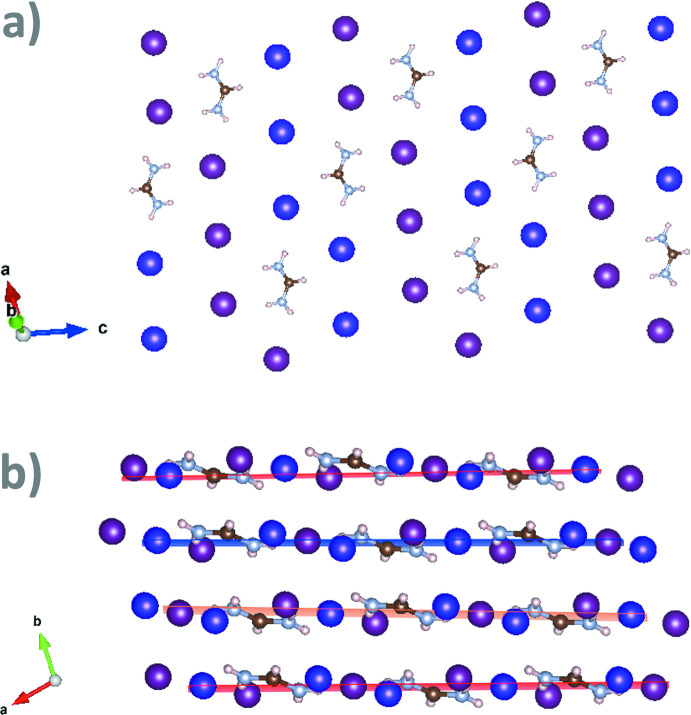
Representation of FAI_3_ as a deformed cubic close-packed crystal structure with both iodine and formamidinium units in the close-packing layer. Purple and violet atoms represent positions of iodine from the first and second types of I_3_^−^, respectively. Formamidinium cations are decreased in size for clarity. (*a*) A single close-packed layer and (*b*) representation of FAI_3_ as a deformed three-layered close-packed structure (the different types of alternating close-packed layers are shown in red, orange and blue).

Besides, the FAI_3_ structure can be represented as a pseudocubic close-packed structure with both iodine and formamidinium units in the close-packing layers (Fig. 3 ▸). In the discussed crystal structure, each center of mass of the formamidinium cation and each iodine have 12 neighbors in the first coordination sphere, resulting in a distorted cubocta­hedra occupancy, which is typical for pseudocubic close-packing. In comparison, the formamidinium iodide structure can be described as a pseudohexa­gonal close-packed structure with both iodine and formamidinium units in the close-packing layers (Petrov, Goodilin *et al.*, 2017 ▸).

## Synthesis and crystallization   

FAI and I_2_ were purchased from Dyesol (99.9% purity) and Ruskhim (99% purity) without further purification. To obtain single crystalline **I**, the stoichiometric FAI/I_2_ mixture was previously powdered in dry air glovebox. After that, the powdered mixture was slowly heated up to 355–358 K and the obtained single crystals were used for the refinement of crystal structure.

## Refinement   

Crystal data, data collection and structure refinement details are summarized in Table 3 ▸. All H atoms were found in an electron density-difference map and refined with isotropic displacement parameters. The crystal studied was refined as a three-component twin with domain ratios of 0.631 (1):0.211 (1):0.158 (1). The second and third major domains are rotated from the first one by ∼180° about reciprocal axes [101] and [110], respectively.

## DFT calculations   

The electronic structure of the crystal FAI_3_ was calculated using the DMol3 module from the *Materials Studio* software package (Delley, 2000 ▸, 1990 ▸). In the applied DFT method, the PBE functional was used with the DNP 4.4 (double numerical plus polarization) basis set of atomic functions with all electron relativistic core treatment. The charges (Table 4 ▸) were derived according to Mulliken’s scheme. The calculations were performed without further optimization.

Computations of Bader atomic charges were performed in the *GAUSSIAN 09* program (Frisch *et al.*, 2016 ▸) using density functional theory (PBE0) (Perdew *et al.*, 1996 ▸) and the def-2-TZVP basis set. The geometry was optimized using the very tight optimization criteria and empirical dispersion corrections on the total energy (Grimme *et al.*, 2010 ▸) with Becke–Johnson damping (D3) (Grimme *et al.*, 2011 ▸).

Topological analysis of the ρ(**r**) function, calculations of the v(**r_bcp_**) and integration over inter­atomic zero-flux surfaces were performed using the *AIMAll* program (Keith, 2013 ▸).

## Supplementary Material

Crystal structure: contains datablock(s) I. DOI: 10.1107/S2056989021005673/dx2037sup1.cif

Structure factors: contains datablock(s) I. DOI: 10.1107/S2056989021005673/dx2037Isup2.hkl

CCDC reference: 2087353

Additional supporting information:  crystallographic information; 3D view; checkCIF report

## Figures and Tables

**Table 1:** Hydrogen-bond geometry (Å, °) and calculated charges for the specified hydrogen atom derived according to the Mulliken scheme

*D*—H⋯*A*	*D*—H	H⋯*A*	*D*⋯*A*	*D*—H⋯*A*	H charge
C1—H1*A*⋯I4^i^	0.95	3.03	3.80 (2)	138	0.152
N1—H1*B*⋯I2^ii^	0.88	3.01	3.73 (2)	140	0.164
N1—H1*C*⋯I4^iii^	0.88	2.92	3.74 (2)	155	0.160
N2—H2*A*⋯I2	0.88	2.81	3.65 (2)	161	0.171
N2—H2*B*⋯I2^iv^	0.88	3.08	3.609 (19)	121	0.189
N2—H2*B*⋯I4^v^	0.88	2.94	3.66 (2)	140	0.189

Symmetry codes: (i) −*x* + 1, −*y* + 1, −*z* + 1; (ii) −*x* + 1, −*y*, −*z* + 1; (iii) *x* − 1, *y* − 1, *z* − 1; (iv) −*x*, *y*, *z* − 1; (v) −*x* + 2, −*y* + 1, −*z* + 1.

**Table 2:** Calculated Bader atomic charges for the isolated symmetric formamid­inium cation. The order of the atoms in the isolated cation matches with that in the formamidinium cation in the refined crystal structure

C1	+1.31	H1*C*	+0.50
H1*A*	+0.18	N2	−1.23
N1	−1.23	H2*A*	+0.48
H1*B*	+0.48	H2*B*	+0.50

**Table 3:** Experimental details

Crystal data	
Chemical formula	CH_5_N_2_^+^·I_3_^−^
*M*_r_	425.77
Crystal system, space group	Triclinic, *P*\overline{1}
Temperature (K)	110
*a*, *b*, *c* (Å)	6.0767 (10), 6.1886 (11), 11.727 (2)
α, β, γ (°)	97.512 (6), 100.345 (6), 99.437 (6)
*V* (Å^3^)	422.10 (13)
*Z*	2
Radiation type	Mo *K*α
μ (mm^−1^)	11.01
Crystal size (mm)	0.26 × 0.19 × 0.08

Data collection	
Diffractometer	Bruker D8 Quest with Photon III detector
Absorption correction	Multi-scan (*TWINABS*; Bruker, 2019 ▸)
*T*_min_, *T*_max_	0.044, 0.092
No. of measured, independent and observed [*I* > 2σ(*I*)] reflections	4015, 4015, 2217
(sin θ/λ)_max_ (Å^−1^)	0.682

Refinement	
*R*[*F*^2^ > 2σ(*F* ^2^)], *wR*(*F* ^2^), *S*	0.084, 0.200, 0.98
No. of reflections	4015
No. of parameters	60
No. of restraints	6
H-atom treatment	H-atom parameters constrained
Δρ_max_, Δρ_min_ (e Å^−3^)	2.45, −2.52

Computer programs: *APEX3* and *SAINT* (Bruker, 2019 ▸), *SHELXT* (Sheldrick, 2015*a* ▸), *SHELXL2018* (Sheldrick, 2015*b* ▸) and *SHELXTL* (Sheldrick, 2008 ▸).

**Table 4:** Atomic charges calculated using Mulliken population analysis

C1	+0.003	I2	−0.388
H1*A*	+0.152	N2	−0.049
N1	+0.019	H2*A*	+0.171
H1*B*	+0.164	H2*B*	+0.189
H1*C*	+0.160	I3	−0.044
I1	−0.030	I4	−0.383

## supplementary crystallographic information

### Crystal data

**Table:**

CH_5_N_2_^+^·I_3_^−^	*Z* = 2
*M**_r_* = 425.77	*F*(000) = 368
Triclinic, *P*1	*D*_x_ = 3.350 Mg m^−^^3^
*a* = 6.0767 (10) Å	Mo *K*α radiation, λ = 0.71073 Å
*b* = 6.1886 (11) Å	Cell parameters from 899 reflections
*c* = 11.727 (2) Å	θ = 3.5–29.1°
α = 97.512 (6)°	µ = 11.01 mm^−^^1^
β = 100.345 (6)°	*T* = 110 K
γ = 99.437 (6)°	Block, red
*V* = 422.10 (13) Å^3^	0.26 × 0.19 × 0.08 mm

### Data collection

**Table:**

Bruker D8 Quest with Photon III detector diffractometer	4015 independent reflections
Radiation source: micro-focus sealed X-ray tube	2217 reflections with *I* > 2σ(*I*)
Detector resolution: 7.31 pixels mm^-1^	θ_max_ = 29.0°, θ_min_ = 1.8°
φ and ω shutterless scans	*h* = −8→8
Absorption correction: multi-scan (*TWINABS*; Bruker, 2019)	*k* = −8→8
*T*_min_ = 0.044, *T*_max_ = 0.092	*l* = 0→15
4015 measured reflections	

### Refinement

**Table:**

Refinement on *F*^2^	Primary atom site location: structure-invariant direct methods
Least-squares matrix: full	Hydrogen site location: inferred from neighbouring sites
*R*[*F*^2^ > 2σ(*F*^2^)] = 0.084	H-atom parameters constrained
*wR*(*F*^2^) = 0.200	*w* = 1/[σ^2^(*F*_o_^2^) + (0.0803*P*)^2^ + 4.4757*P*] where *P* = (*F*_o_^2^ + 2*F*_c_^2^)/3
*S* = 0.98	(Δ/σ)_max_ < 0.001
4015 reflections	Δρ_max_ = 2.45 e Å^−^^3^
60 parameters	Δρ_min_ = −2.52 e Å^−^^3^
6 restraints	

### Special details

**Table:**

Geometry. All esds (except the esd in the dihedral angle between two l.s. planes) are estimated using the full covariance matrix. The cell esds are taken into account individually in the estimation of esds in distances, angles and torsion angles; correlations between esds in cell parameters are only used when they are defined by crystal symmetry. An approximate (isotropic) treatment of cell esds is used for estimating esds involving l.s. planes.
Refinement. Refined as a three-component twin

### Fractional atomic coordinates and isotropic or equivalent isotropic displacement parameters (Å^2^)

**Table:**

	*x*	*y*	*z*	*U*_iso_*/*U*_eq_	
I1	1.000000	0.000000	0.500000	0.0261 (5)	
C1	0.515 (3)	0.307 (3)	0.216 (2)	0.023 (4)	
H1A	0.481532	0.352625	0.141658	0.027*	
N1	0.372 (4)	0.157 (3)	0.238 (2)	0.051 (7)	
H1B	0.396378	0.110694	0.306643	0.061*	
H1C	0.246338	0.097725	0.185971	0.061*	
I2	0.7214 (3)	0.2999 (2)	0.58692 (14)	0.0318 (5)	
N2	0.705 (3)	0.406 (4)	0.2867 (17)	0.037 (5)	
H2A	0.741417	0.369243	0.356493	0.044*	
H2B	0.796631	0.510952	0.264606	0.044*	
I3	1.000000	0.500000	1.000000	0.0258 (5)	
I4	0.7825 (3)	0.8407 (2)	1.10514 (14)	0.0313 (4)	

### Atomic displacement parameters (Å^2^)

**Table:**

	*U*^11^	*U*^22^	*U*^33^	*U*^12^	*U*^13^	*U*^23^
I1	0.0268 (11)	0.0239 (9)	0.0270 (11)	0.0069 (8)	0.0049 (8)	0.0001 (9)
C1	0.020 (6)	0.023 (6)	0.026 (7)	0.009 (5)	0.006 (5)	−0.002 (5)
N1	0.047 (14)	0.029 (11)	0.070 (18)	0.005 (10)	0.004 (12)	−0.003 (12)
I2	0.0324 (8)	0.0297 (8)	0.0338 (10)	0.0113 (6)	0.0084 (7)	−0.0012 (7)
N2	0.036 (12)	0.055 (13)	0.025 (11)	0.009 (10)	0.015 (9)	0.011 (10)
I3	0.0297 (11)	0.0210 (9)	0.0276 (11)	0.0071 (8)	0.0067 (8)	0.0033 (8)
I4	0.0377 (9)	0.0262 (7)	0.0328 (9)	0.0128 (6)	0.0107 (7)	0.0018 (7)

### Geometric parameters (Å, º)

**Table:**

I1—I2	2.9165 (14)	N1—H1C	0.8800
I1—I2^i^	2.9165 (14)	N2—H2A	0.8800
C1—N1	1.25 (3)	N2—H2B	0.8800
C1—N2	1.30 (3)	I3—I4	2.9209 (14)
C1—H1A	0.9500	I3—I4^ii^	2.9209 (14)
N1—H1B	0.8800		
			
I2—I1—I2^i^	180.0	H1B—N1—H1C	120.0
N1—C1—N2	125 (3)	C1—N2—H2A	120.0
N1—C1—H1A	117.3	C1—N2—H2B	120.0
N2—C1—H1A	117.3	H2A—N2—H2B	120.0
C1—N1—H1B	120.0	I4—I3—I4^ii^	180.0
C1—N1—H1C	120.0		

Symmetry codes: (i) −*x*+2, −*y*, −*z*+1; (ii) −*x*+2, −*y*+1, −*z*+2.
